# EMOTIONAL PROCESSING IN ANOREXIA NERVOSA - WHAT IS THE ROLE OF NEUROMODULATION?

**DOI:** 10.1192/j.eurpsy.2023.1111

**Published:** 2023-07-19

**Authors:** C. Pinheiro Ramos, A. R. Vaz, A. Sampaio

**Affiliations:** 1 Faculty of Medicine, University of Lisbon, Lisbon; 2 School of Psychology, University of Minho, Braga, Portugal

## Abstract

**Introduction:**

Anorexia Nervosa (AN) is an Eating Disorder (ED), being globally characterized by a low body mass index (BMI), intense fear of gaining weight, and distorted body image that motivates extreme food restrictions. The consequence is massive weight loss.

AN is the third leading cause of chronic illness among adolescents and the leading cause of death among psychiatric conditions.

Among patients with AN it is common the occurrence of psychiatric comorbidities, particularly depressive and anxiety syndromes.

Negative emotions are very common and represent either primary aspects of the disease or arise secondarily to psychopathological or organic processes.

The therapeutic options for AN are scarce and only work for a small percentage of subjects.

It is known that difficulty in emotional regulation is one of the defining characteristics of ED, being a core feature of AN psychopathology.

**Objectives:**

To highlight the importance of understanding the neurobiology of AN, how it is related to emotional processing and future directions for AN´s management.

**Methods:**

Non-systematic review of the literature using *Pubmed* database. Papers were selected according to their relevance.

**Results:**

In recent literature, in purging AN-type (neurobiology similar to Bulimia Nervosa - BN), binge eating is a method of emotional regulation, while in restricting AN-type, food restriction is the way to deal with emotions, mainly negative emotions.

It is known that in AN, patients tend to eat less than usual in response to a negative emotion and more than usual in response to a positive emotion. In BN, the neurobiology works in a mirrored way, patients eat less than usual in response to a positive emotion and more than usual in response to a negative emotion.

In short, in the face of negative emotions, subjects with AN respond with dietary restriction and, subjects with BN respond with binge eating. On the other hand, more positive emotions seem to resolve the maladaptive eating behaviours inherent to both ED, with AN and BN subjects tending towards more balanced eating behaviours.

One of the brain areas most implicated in the neurobiology of AN is the left dorsolateral prefrontal cortex (L-DLPFC), since this region is recognized as being involved in decision-making process and emotional regulation, and is therefore the target of novel and experimental treatment strategies, namely those related with neuromodulation, particularly Transcranial Magnetic Stimulation (TMS).

**Conclusions:**

Emotional regulation, particularly the processing of negative emotions, appears to be a key element in the neurobiology of AN.

With new neuromodulation techniques, specially TMS, it seems possible to modulate the neuronal circuits inherent to emotional processing, such as the L-DLPFC.

Future randomized clinical trials are needed in order to understand how neuromodulation can contribute to exploring the neurobiology of AN and to become more targeted and effective therapeutic options.

**Disclosure of Interest:**

None Declared

